# *In silico* reconstitution of DNA replication. Lessons from single-molecule imaging and cryo-tomography applied to single-particle cryo-EM

**DOI:** 10.1016/j.sbi.2021.11.015

**Published:** 2022-02

**Authors:** Julia F. Greiwe, Giulia Zanetti, Thomas C.R. Miller, Alessandro Costa

**Affiliations:** 1 Macromolecular Machines Laboratory, The Francis Crick Institute, London NW1 1AT, UK; 2 Institute of Structural and Molecular Biology, Birkbeck College, Malet St., London WC1E 7HX, UK; 3 Center for Chromosome Stability, Department of Cellular and Molecular Medicine, University of Copenhagen, Blegdamsvej 3B, 2200 Copenhagen N, Denmark

**Keywords:** COPII, Coat Protein Complex II, CoSMoS, Co-localization Single Molecule Specroscopy, Cryo-EM, Cryo-electron Microscopy, MCM, MiniChromosome Maintenance, ORC, Origin Recognition Complex, TIRF, Total Internal Reflection Fluorescence

## Abstract

DNA replication has been reconstituted *in vitro* with yeast proteins, and the minimal system requires the coordinated assembly of 16 distinct replication factors, consisting of 42 polypeptides. To understand the molecular interplay between these factors at the single residue level, new structural biology tools are being developed. Inspired by advances in single-molecule fluorescence imaging and cryo-tomography, novel single-particle cryo-EM experiments have been used to characterise the structural mechanism for the loading of the replicative helicase. Here, we discuss how *in silico* reconstitution of single-particle cryo-EM data can help describe dynamic systems that are difficult to approach with conventional three-dimensional classification tools.

## Introduction

Biochemical reconstitution using purified proteins has been used to recapitulate a wide array of biological processes *in vitro*, ranging from nuclear DNA replication to vesicle trafficking between cellular compartments [1, 2, 3, 4]. Biological imaging is now faced with the task of describing the dynamic interplay between individual factors that cooperate to perform these complex multicomponent reactions. Three approaches are providing critical contributions. *i.* Single-particle cryo electron microscopy (cryo-EM) can yield atomic, or near-atomic, resolution views of individual factors caught in the act of performing their biological function [5, 6, 7]. *ii.* Cryo-tomography is used to describe molecular ultrastructures in their physiologically relevant environment [8]. *iii.* Single-molecule fluorescence microscopy allows real-time tracking of molecular co-localisation and long-range movements, useful to establish the order of molecular events in a biological pathway [9]. In this article, we review the tools available to investigate the structural dynamics underlying molecular mechanisms for reconstituted multicomponent reactions. Focussing on the example of eukaryotic DNA replication reconstituted *in vitro*, we discuss the underexploited potential of single-particle cryo-EM for describing molecular mechanisms in a broad, physiological context, at the single-residue level. Currently, the architecture of dynamic reconstituted systems can be intractable even with modern image processing tools developed to handle structural heterogeneity [10∗, 11, 12]. However, by adapting approaches from cryo-tomography and single-molecule imaging for single-particle analysis, we argue that the molecular ultrastructure of dynamic multicomponent reactions can be resolved to describe complex molecular mechanisms.

## Ultrastructural understanding of a reconstituted process: the example of DNA replication

**Figure 1:**
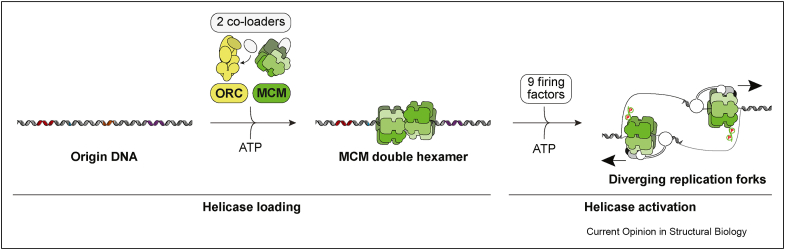
Eukaryotic replicative helicase loading and activation at an origin of replication. Aided by two co-loaders, ORC binds to origin DNA and recruits two ring-shaped MCM helicases, forming a head-to-head double hexamer. The double hexamer is catalytically inactive, and a set of 9 firing factors switch on the DNA unwinding function of MCM, in a process that requires a set of ATP binding, phosphorylation and ATP hydrolysis events. On activation, the double hexamer is broken, and two helicase particles translocate in opposite directions, exposing the single-stranded DNA template for the replicative polymerases.

Eukaryotic DNA replication has been reconstituted *in vitro* using purified yeast proteins. The minimal set of components required to achieve replication include 16 purified factors made from a total of 42 polypeptides that perform functions including phosphorylation, ATP hydrolysis and nucleotide polymerisation [1]. DNA replication requires a helicase that unwinds the DNA and exposes the single-stranded DNA templates for dedicated replicative polymerases [2,13,14]. The hexameric ring-shaped replicative helicase (named minichromosome maintenance or MCM complex) is first loaded onto replication start sites (origins), by the origin recognition complex (ORC) and other co-loaders [15]. This process results in pairs of MCM rings encircling duplex DNA at each origin, with each pair forming a symmetric double hexamer that remains catalytically inactive until the synthesis (S) phase of the cell cycle [16, 17, 18, 19, 20] (Figure 1). Nine firing factors are required to activate the helicase, splitting the double hexamer into two translocating DNA-unwinding particles that open a replication bubble by moving in opposite directions [1,21]. Long-standing questions in the field include *i.* how ORC discriminates origin recognition sites, *ii.* how two DNA-loaded helicases are assembled into a double hexamer that contains the symmetry required for bidirectional replication and *iii.* the directionality of movement of the activated helicase.

Single-particle studies provided the near-atomic resolution structure of several isolated DNA replication factors [22]. This led to identifying, for example, individual amino acid residues involved in the sequence-specific DNA recognition that allows yeast ORC to target origin DNA [23]. Single-particle structures are static, however, and isolated from their physiological context. As such, following the sequence of molecular events leading to helicase loading via single-molecule fluorescence microscopy has been important, to understand the dynamic interactions between the MCM helicase and its loaders over time [24∗∗, 25, 26, 27∗, 28]. To understand the broader context of the helicase loading reaction, describing the relative orientation of helicases and loading factors bound to the same stretch of DNA was also critical. Cryo-tomography studies have spearheaded developments to integrate structural averages into their native environment captured in three-dimensional (3D) tomograms [8]. Inspired by these achievements, single-particle studies can now be designed to address structural mechanisms of DNA replication and other processes, by integrating high-resolution structures obtained from time-resolved experiments with information from their reconstituted molecular context [29].

## Lessons from single-molecule fluorescence imaging

**Figure 2:**
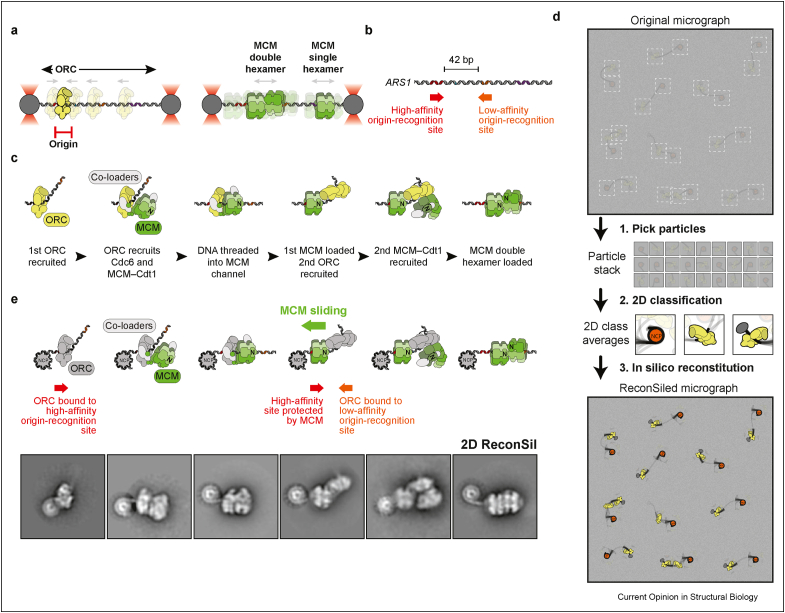
Origin recognition and helicase loading described using single-molecule fluorescence and single-particle cryo-EM. (**a**) Confocal microscopy imaging of origin DNA trapped by optical tweezers shows that ORC diffuses linearly along a DNA segment until the origin sequence is identified. (**b**) Linear structure of the ARS1 origin of replication. (**c**) The sequence of events leading to double hexamer formation. *i.* ORC binds and bends DNA. *ii.* ORC recruits the first MCM ring by binding its C-terminal face. *iii.* DNA is threaded into the central channel of MCM. *iv.* ORC binds to the N-terminal face of the MCM ring. *v.* ORC recruits a second MCM ring by interacting with the same C-terminal face as the first ring. *vi.* The MCM double hexamer is loaded around duplex DNA. (**d**) Cartoon representation of the *in silico* reconstitution (ReconSil) procedure. Particles are picked from a micrograph with a low signal-to-noise ratio. 2D averages are calculated. Averages are overlaid to the original particles in the raw micrographs. Proximal averages that are bound to the same DNA segment are extracted, recovering the full nucleoprotein context at an origin of replication. NCP—nucleosome core particle. (**e**) ReconSil reveals that on the ARS1 origin, the first loaded MCM ring must slide to expose the inverted lower affinity ORC site (orange), hence driving its ORC recognition. This occurs concomitantly with the engagement of the N-terminal face of MCM. By sliding along DNA, the first loaded MCM creates enough space for two sequential ORC binding events that drive double-hexamer formation.

Fluorescence microscopy has been used to track helicase loading and DNA duplication reactions and their compositional dynamics over time on defined DNA sequences [24∗∗, 25, 26, 27∗, 28]. For example, in a confocal microscopy experiment, bead-tethered duplex DNA captured with optical tweezers was used to track the helicase loader ORC, in the process of recognising an origin of replication [24]. Yeast ORC was observed to diffuse linearly along a DNA segment of defined length, sequence and polarity and stop when the origin sequence was found, coherent with previous observations by DNA curtain total internal reflection fluorescence (TIRF) microscopy [28] (Figure 2a). MCM loading by the ORC resulted in the DNA association of double hexamers that are known to be competent for replication. Using this highly sensitive single-molecule assay, loaded single hexamers, which result from a transient interaction with ORC, were also observed [24,26]. As loaded single hexamers are known to be incompetent for replication [30], this finding could explain the observation that many more MCM molecules are loaded onto origin DNA in cells than are actually used during replication [31,32]. Both single as well as double hexamers were observed to slowly diffuse away from the origin site and move linearly along the bead-trapped DNA, compatible with previous biochemical, cellular and structural observations [16,17,21,33]. These results serve as example for how tracking origin recognition and helicase loading reactions over time and along a defined DNA molecule can increase our understanding of molecular mechanisms [24].

In another study, co-localisation single-molecule spectroscopy (CoSMoS) was used to define the order of molecular events leading to double hexamer formation, revealing that, during this process, the loading of the first and second hexameric ring is sequential. Two different sets of co-loaders facilitate the association of the first and second MCM hexamer with DNA [26]. In this context, co-loader release is required for closing each MCM ring around DNA [25]. In contrast, one single ORC molecule was observed to be sufficient during the loading of both MCM rings to form a double hexamer, suggesting that the first loaded hexamer serves to recruit the second hexamer onto DNA [26]. In summary, fluorescence co-localisation experiments established the sequential steps in the helicase loading mechanism.

## Single-particle cryo-EM characterisation of the double-hexamer loading reaction

The CoSMoS observation that one single ORC molecule is sufficient to complete double hexamer loading appeared to be at odds with biochemical evidence supporting a two-ORC mechanism. The two-ORC model was based on data showing that loading of the first and second helicase ring requires the same MCM element and two separate ORC binding events at inverted origin recognition sites to support efficient double-hexamer formation [34,35]. To understand how the one-ORC and two-ORC models could be reconciled, we sought to visualise double hexamer loading at near-atomic resolution while it was occurring *in vitro*. To this end, we took inspiration from single molecule studies to design a time-resolved cryo-EM experiment. As a DNA substrate, we chose the well-characterised yeast ARS1 origin sequence, which contains a higher and a lower affinity origin recognition site (Figure 2b) [36], capped by recognisable, asymmetric roadblocks (either a nucleosome or a covalently linked methyltransferase adduct) [29]. These roadblocks would serve to both retain topologically loaded helicases on the DNA by preventing them from sliding off, but also function as markers that would orient the origin-associated factors with respect to the origin DNA sequence. We then assembled the helicase loading reaction and prepared grids for negative stain electron microscopy at different time points (2–30 minutes). As time progressed, the single-particle count of the loading-competent form of MCM decreased, whereas the double-hexamer species increased. Thus, it was possible to follow double-hexamer loading by time-resolved electron microscopy using sampling rates on a minute time scale. At early time points, we observed the accumulation of two helicase loading intermediates. One showed ORC contacting the C-terminal face of one MCM ring, as previously observed in cryo-EM studies of helicase loading stalled by using a slowly hydrolysable nucleotide analogue (third intermediate in Figure 2c). The structure shows MCM encircling (although not fully locked around) duplex DNA [37,38]. This molecular species disappeared at later time points in our reaction, providing strong evidence that ORC contacting the C-terminal face of MCM is a bona fide loading intermediate. The second molecular species in our time-resolved experiment showed ORC in a completely different configuration and interacting with the N-terminal face of the MCM ring [29]. The corresponding cryo-EM structure showed an MCM hexamer locked around duplex DNA, with ORC engaging a site on the N-terminal face of the MCM that only exists when the helicase ring is fully closed. In this configuration, the DNA-bound ORC is in an inverted orientation compared with the C-terminally interacting ORC (fourth intermediate in Figure 2c). Deeper single-particle cryo-EM analysis also revealed that the N-terminally interacting ORC can recruit a second MCM hexamer via the same mechanism as the first hexamer (fifth intermediate in Figure 2c).

Collectively, these data revealed that the previously published CoSMoS [26] and biochemical [34,35] experiments were not describing two separate mechanisms but rather two distinct aspects of the same process. As in the CoSMoS experiment, first and second hexamer loading was observed to be sequential [26]. The first loaded MCM served to recruit the second MCM molecule, via the previously unrecognised ORC interaction at the N-terminal face of the first loaded hexamer [29]. Coherent with biochemical evidence, this interaction involves two distinct ORC-DNA binding events involving inverted origin recognition sites, with the first and second hexameric rings recruited via the same mechanism [34,35].

Although these results were informative, one critical aspect of our model was not addressed using conventional single-particle analysis. In the yeast ARS1 origin, the proximity of the two inverted ORC binding sites means that the first loaded helicase initially occupies the secondary ORC binding site. To generate enough space for double-hexamer formation, the first loaded MCM must be able to slide along the DNA to allow the N-terminally interacting ORC to reach the second inverted origin recognition site [39]. Because of the limited persistence length of DNA, the entire origin was too flexible to be fully represented in one averaged structure, meaning that a high-resolution view of our new, critical helicase loading intermediate could not be visualised in the full context of the origin [29]. It was thus possible to characterise MCM–ORC interactions on duplex DNA, but not orient the structure with respect to the origin DNA sequence. To address this issue, a reconstitution *in silico* (ReconSil) approach was developed, aimed at generating high-signal views of complete, individual origins of replication with associated helicase loading intermediates [29]. This strategy was inspired by cryo-tomography approaches [8], further discussed in the following. After particle picking and extensive two-dimensional (2D) averaging, helicase loading intermediates were selected, along with nucleosomes capping the DNA substrate in proximity to the (higher-affinity) ORC binding site [40]. By combining coordinates derived from particle picking with translations and rotations applied during 2D classification, 2D classes were overlayed onto their constituent particles in the raw micrographs. Neighbouring particles that co-localised to a single DNA stretch were then selected, which allowed the recovery of complete origin images (Figure 2d). The nucleosome, designed to cap one end of the origin DNA, served to orient the MCM loading intermediates with respect to the origin sequence. ReconSil led to the discovery that the first loaded MCM slides along DNA to concomitantly occupy the high-affinity origin recognition site and expose the lower-affinity site, hence facilitating the transition from a C-terminally to an N-terminally interacting ORC, with inverted polarity of DNA binding [29] (Figure 2e). Simple in its implementation, ReconSil proved to be a powerful tool to describe extremely flexible assemblies that are otherwise intractable, even by modern tools developed to handle structural heterogeneity.

## Lessons from cryo-tomography and sub-tomogram averaging

The idea of positioning structural averages back into the image that raw particles were cropped out of was first implemented in cryo-tomography [41], which is considered the technique of choice for studying macromolecular assemblies in their native environment [42]. This approach led to describing cellular and viral ultrastructures *in situ* or highly structured reconstituted systems, as, for example, the COPII vesicle budding apparatus [43,44]. Cryo-electron tomograms are 3D volumes reconstructed by back-projecting tilted images of the same field of view. They can be difficult to interpret because of low signal and anisotropic resolution, but averaging of cropped subtomograms yields interpretable structures [8]. Indeed, pioneering studies using optimised imaging conditions and improved computational tools demonstrated that near-atomic resolution structures are achievable [43,45,46]; however, this is still challenging for asymmetric complexes that are smaller than 1 MDa or do not form a structured array [47,48]. Much like the single-particle ReconSil implementation described previously, a broader physiological context can be reconstituted when subtomogram averages are placed back into the cryo-tomograms, retrieving information on the relative orientation of individual particles [41], which can be seen as individual bricks of a molecular ultrastructure.

**Figure 3:**
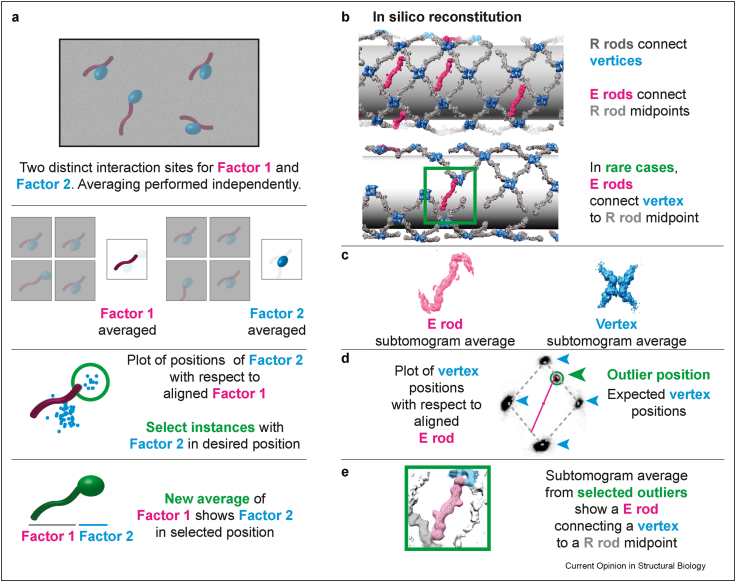
*In silico* reconstitution of supramolecular assemblies, lessons from cryo-tomography. (**a**) Cartoon representation of low signal-to-noise tomogram of factor 1 and factor 2 connected via two distinct interfaces. In the class average centred on factor 1, factor 2 is averaged out and vice versa. The position of factor 2 can be plotted with respect to aligned factor 1, which identifies clusters with two discrete orientations. Factor 1 particles can be subclassified as per the interaction clusters and subtomograms from these discrete classes can be extracted and used to generate a new 3D average where both factor 1 and factor 2 are resolved. (**b**–**e**) An example from cryo-tomography of the COPII outer coat. (**b**) Four ‘R’ rods (grey) interact to form a rhomboidal pattern. Extra (‘E’) rods (purple) connect R rod midpoints. Rarely, an E rod connects a rhomboid vertex (blue) with the midpoint of a neighbouring R rod. (**c**) Subtomogram averages of E rods and vertices. (**d**) The position of vertices is plotted with respect to the aligned E rods. Clustering reveals the expected rhomboidal pattern and also one outlier position. (**e**) Selecting E rods from the outlier cluster reveals a noncanonical interaction, with the E rod connecting a vertex to the midpoint of a neighbouring R rod.

Plotting the relative orientations of individual particles can also help characterize poorly populated yet discrete interactions that are difficult to identify using traditional 3D classification methods (Figure 3a). This approach was introduced in a recent cryo-tomography study on *in vitro* reconstituted vesicle budding to identify previously unreported interactions in the yeast COPII lattice that scaffolds membrane curvature [43]. The COPII coat is composed of two concentric layers around a membrane. These layers have different compositions that can be separately resolved from reconstituted coated vesicles using targeted subtomogram averaging [43,44,49]. The outer layer is composed of Sec13-31 rods that meet at vertices to create a rhomboidal cage-like structure. Extra (‘E’) rods connect the midpoint of two rhomboidal (‘R’) rods. Plotting the relative positions of Sec13/Sec31 vertices with respect to the aligned E rods led to visualising the expected rhomboidal lattice [44], but also allowed the observation of an anomalous cluster of neighbouring vertices, revealing previously unreported interactions. In this outlier cluster, E rods connect a vertex at one end and with the midpoint of an R rod at the other end (Figure 3b). After selecting the subtomograms corresponding to the outlier cluster, a 3D average could be determined, confirming the existence of two distinct connectivities in the COPII lattice (Figure 3c–e) [43]. Statistical analysis on neighbouring particles can therefore serve as an alternative strategy to identify rare discrete super-molecular assemblies that escape 3D classification. Adapting this approach to single-particle ReconSil promises to be a powerful tool for the ultrastructural characterisation of complex multicomponent reactions such as reconstituted DNA replication.

## Conclusions and future perspectives

The reconstitution of origin-dependent DNA replication using purified yeast proteins has changed the scale of mechanistic questions that can now be addressed using single-particle cryo-EM. For example, the processes of origin-DNA melting and replication-fork establishment can now be investigated, alongside replication through chromatin and the mechanism of parental histone redeposition at the basis of epigenetic inheritance.

Time-resolved cryo-EM studies are an exciting developing area in this context. Although we demonstrated that DNA loading of the MCM helicase can be studied by recording time points with minute resolution [29], other structural transitions on the path to origin activation will likely require higher sampling frequencies. One example is the transition from the duplex interacting to the single-stranded DNA interacting form of the MCM, which occurs on establishment of the replication fork [21]. New technology for rapid mixing using modular microfluidics and blot-free vitrification in the millisecond resolution time scale will likely be useful to achieve these tasks [50∗∗, 51∗, 52].

The combination of high-resolution time-resolved single-particle EM with *in silico*–reconstitution approaches promises to provide a valuable tool to study dynamic and heterogeneous ultrastructures. As ReconSil has so far only been implemented with 2D averages and not 3D structures, it bypasses the requirement for the tilt-series acquisition required for cryo-tomography, yet it applies the logic of particle repositioning into the raw image (2D micrograph) to reconstitute the full context of a given reaction [29]. Future 3D implementations will be useful to describe complex and flexible ultrastructures at single-particle resolution. 3D structures obtained by single-particle reconstruction can be repositioned into the locations obtained from raw micrographs, starting from the x and y coordinates of individual picked particles. Individual 2D micrographs, however, contain no information along the z axis. For DNA replication reactions, this means that repositioned 3D structures bound to the same DNA segment would not be aligned along the z axis, unless DNA lies on a plane parallel to the image detector. Retrieving accurate z-axis information will be key for the robust implementation ReconSil in three dimensions. To achieve this, tilt-pair acquisition could be used [53]. In more sophisticated, recently developed approaches, 3D refinement, as implemented in single-particle analysis, could be applied to cryo-tomographic tilt series at the single sub-tomogram level. Here, a 3D volume is refined from tilted images of single particles from individual sub-tomograms, using constraints derived from the tilt-series acquisition [46,54,55]. In a different implementation, a high-dose nontilted image is acquired, followed by a tilt series of the same field of view. A sub-tomogram average is first computed from the tilt series, whose resolution is limited because of over-exposure and the errors in cryo-tomogram reconstruction, yet it is useful to generate initial alignment constraints. A high-resolution structure is then computed using the nontilted, uncorrupted image, after imposing the subtomogram constraints [56,57]. Applied to ReconSil, these approaches could help align repositioned 3D volumes along the z axis, to recover optimally aligned factors bound to a continuous DNA segment. Here, we have focused our discussion on how the combination of time-resolved and *in silico* reconstitution approaches can help describe the molecular mechanisms of DNA replication. However, in the future, we envisage that these approaches will have wide applicability to other areas of biology where discrete factors act on shared molecular substrates or scaffolds.

## Conflict of interest statement

Nothing declared.

## References

[1] Yeeles J.T., Deegan T.D., Janska A., Early A., Diffley J.F. (2015). Regulated eukaryotic DNA replication origin firing with purified proteins. Nature.

[2] Yeeles J.T., Janska A., Early A., Diffley J.F. (2017). How the eukaryotic replisome achieves rapid and efficient DNA replication. Mol Cell.

[3] Bacia K., Futai E., Prinz S., Meister A., Daum S., Glatte D., Briggs J.A., Schekman R. (2011). Multibudded tubules formed by COPII on artificial liposomes. Sci Rep.

[4] Faini M., Prinz S., Beck R., Schorb M., Riches J.D., Bacia K., Brugger B., Wieland F.T., Briggs J.A. (2012). The structures of COPI-coated vesicles reveal alternate coatomer conformations and interactions. Science.

[5] Nogales E., Scheres S.H. (2015). Cryo-EM: a unique tool for the visualization of macromolecular complexity. Mol Cell.

[6] Nakane T., Kotecha A., Sente A., McMullan G., Masiulis S., Brown P., Grigoras I.T., Malinauskaite L., Malinauskas T., Miehling J. (2020). Single-particle cryo-EM at atomic resolution. Nature.

[7] Yip K.M., Fischer N., Paknia E., Chari A., Stark H. (2020). Atomic-resolution protein structure determination by cryo-EM. Nature.

[8] Castano-Diez D., Zanetti G. (2019). In situ structure determination by subtomogram averaging. Curr Opin Struct Biol.

[9] Monachino E., Spenkelink L.M., van Oijen A.M. (2017). Watching cellular machinery in action, one molecule at a time. J Cell Biol.

[bib10] Zhong E.D., Bepler T., Berger B., Davis J.H., CryoDRGN (2021). Reconstruction of heterogeneous cryo-EM structures using neural networks. Nat Methods.

[11] Punjani A., Fleet D.J. (2021). 3D variability analysis: resolving continuous flexibility and discrete heterogeneity from single particle cryo-EM. J Struct Biol.

[12] Nakane T., Kimanius D., Lindahl E., Scheres S.H. (2018). Characterisation of molecular motions in cryo-EM single-particle data by multi-body refinement in RELION. Elife.

[13] Attali I., Botchan M.R., Berger J.M. (2021). Structural mechanisms for replicating DNA in eukaryotes. Annu Rev Biochem.

[14] Georgescu R.E., Schauer G.D., Yao N.Y., Langston L.D., Yurieva O., Zhang D., Finkelstein J., O'Donnell M.E. (2015). Reconstitution of a eukaryotic replisome reveals suppression mechanisms that define leading/lagging strand operation. Elife.

[15] Bleichert F. (2019). Mechanisms of replication origin licensing: a structural perspective. Curr Opin Struct Biol.

[16] Remus D., Beuron F., Tolun G., Griffith J.D., Morris E.P., Diffley J.F. (2009). Concerted loading of Mcm2-7 double hexamers around DNA during DNA replication origin licensing. Cell.

[17] Evrin C., Clarke P., Zech J., Lurz R., Sun J., Uhle S., Li H., Stillman B., Speck C. (2009). A double-hexameric MCM2-7 complex is loaded onto origin DNA during licensing of eukaryotic DNA replication. Proc Natl Acad Sci U S A.

[18] Abid Ali F., Douglas M.E., Locke J., Pye V.E., Nans A., Diffley J.F.X., Costa A. (2017). Cryo-EM structure of a licensed DNA replication origin. Nat Commun.

[19] Noguchi Y., Yuan Z., Bai L., Schneider S., Zhao G., Stillman B., Speck C., Li H. (2017). Cryo-EM structure of Mcm2-7 double hexamer on DNA suggests a lagging-strand DNA extrusion model. Proc Natl Acad Sci U S A.

[20] Li N., Zhai Y., Zhang Y., Li W., Yang M., Lei J., Tye B.K., Gao N. (2015). Structure of the eukaryotic MCM complex at 3.8 A. Nature.

[21] Douglas M.E., Ali F.A., Costa A., Diffley J.F.X. (2018). The mechanism of eukaryotic CMG helicase activation. Nature.

[22] Yuan Z., Li H. (2020). Molecular mechanisms of eukaryotic origin initiation, replication fork progression, and chromatin maintenance. Biochem J.

[23] Li N., Lam W.H., Zhai Y., Cheng J., Cheng E., Zhao Y., Gao N., Tye B.K. (2018). Structure of the origin recognition complex bound to DNA replication origin. Nature.

[bib24] Sanchez H., McCluskey K., van Laar T., van Veen E., Asscher F.M., Solano B., Diffley J.F.X., Dekker N.H. (2021). DNA replication origins retain mobile licensing proteins. Nat Commun.

[25] Ticau S., Friedman L.J., Champasa K., Correa I.R., Gelles J., Bell S.P. (2017). Mechanism and timing of Mcm2-7 ring closure during DNA replication origin licensing. Nat Struct Mol Biol.

[26] Ticau S., Friedman L.J., Ivica N.A., Gelles J., Bell S.P. (2015). Single-molecule studies of origin licensing reveal mechanisms ensuring bidirectional helicase loading. Cell.

[bib27] Lewis J.S., Spenkelink L.M., Schauer G.D., Yurieva O., Mueller S.H., Natarajan V., Kaur G., Maher C., Kay C., O'Donnell M.E. (2020). Tunability of DNA polymerase stability during eukaryotic DNA replication. Mol Cell.

[28] Duzdevich D., Warner M.D., Ticau S., Ivica N.A., Bell S.P., Greene E.C. (2015). The dynamics of eukaryotic replication initiation: origin specificity, licensing, and firing at the single-molecule level. Mol Cell.

[bib29] Miller T.C.R., Locke J., Greiwe J.F., Diffley J.F.X., Costa A. (2019). Mechanism of head-to-head MCM double-hexamer formation revealed by cryo-EM. Nature.

[bib30] Champasa K., Blank C., Friedman L.J., Gelles J., Bell S.P. (2019). A conserved Mcm4 motif is required for Mcm2-7 double-hexamer formation and origin DNA unwinding. Elife.

[31] Crevel G., Hashimoto R., Vass S., Sherkow J., Yamaguchi M., Heck M.M., Cotterill S. (2007). Differential requirements for MCM proteins in DNA replication in Drosophila S2 cells. PLoS One.

[32] Lei M., Kawasaki Y., Tye B.K. (1996). Physical interactions among Mcm proteins and effects of Mcm dosage on DNA replication in Saccharomyces cerevisiae. Mol Cell Biol.

[33] Gros J., Kumar C., Lynch G., Yadav T., Whitehouse I., Remus D. (2015). Post-licensing specification of eukaryotic replication origins by facilitated mcm2-7 sliding along DNA. Mol Cell.

[34] Coster G., Diffley J.F.X. (2017). Bidirectional eukaryotic DNA replication is established by quasi-symmetrical helicase loading. Science.

[35] Frigola J., Remus D., Mehanna A., Diffley J.F. (2013). ATPase-dependent quality control of DNA replication origin licensing. Nature.

[36] Marahrens Y., Stillman B. (1992). A yeast chromosomal origin of DNA replication defined by multiple functional elements. Science.

[37] Sun J., Evrin C., Samel S.A., Fernandez-Cid A., Riera A., Kawakami H., Stillman B., Speck C., Li H. (2013). Cryo-EM structure of a helicase loading intermediate containing ORC-Cdc6-Cdt1-MCM2-7 bound to DNA. Nat Struct Mol Biol.

[38] Yuan Z., Riera A., Bai L., Sun J., Nandi S., Spanos C., Chen Z.A., Barbon M., Rappsilber J., Stillman B. (2017). Structural basis of Mcm2-7 replicative helicase loading by ORC-Cdc6 and Cdt1. Nat Struct Mol Biol.

[39] Warner M.D., Azmi I.F., Kang S., Zhao Y., Bell S.P. (2017). Replication origin-flanking roadblocks reveal origin-licensing dynamics and altered sequence dependence. J Biol Chem.

[40] Lee D.G., Bell S.P. (1997). Architecture of the yeast origin recognition complex bound to origins of DNA replication. Mol Cell Biol.

[41] Pruggnaller S., Mayr M., Frangakis A.S. (2008). A visualization and segmentation toolbox for electron microscopy. J Struct Biol.

[42] Pfeffer S., Mahamid J. (2018). Unravelling molecular complexity in structural cell biology. Curr Opin Struct Biol.

[bib43] Hutchings J., Stancheva V.G., Brown N.R., Cheung A.C.M., Miller E.A., Zanetti G. (2021). Structure of the complete, membrane-assembled COPII coat reveals a complex interaction network. Nat Commun.

[44] Zanetti G., Prinz S., Daum S., Meister A., Schekman R., Bacia K., Briggs J.A. (2013). The structure of the COPII transport-vesicle coat assembled on membranes. Elife.

[45] Schur F.K., Obr M., Hagen W.J., Wan W., Jakobi A.J., Kirkpatrick J.M., Sachse C., Krausslich H.G., Briggs J.A. (2016). An atomic model of HIV-1 capsid-SP1 reveals structures regulating assembly and maturation. Science.

[bib46] Tegunov D., Xue L., Dienemann C., Cramer P., Mahamid J. (2021). Multi-particle cryo-EM refinement with M visualizes ribosome-antibiotic complex at 3.5 A in cells. Nat Methods.

[47] Ke Z., Oton J., Qu K., Cortese M., Zila V., McKeane L., Nakane T., Zivanov J., Neufeldt C.J., Cerikan B. (2020). Structures and distributions of SARS-CoV-2 spike proteins on intact virions. Nature.

[48] Turonova B., Sikora M., Schurmann C., Hagen W.J.H., Welsch S., Blanc F.E.C., von Bulow S., Gecht M., Bagola K., Horner C. (2020). In situ structural analysis of SARS-CoV-2 spike reveals flexibility mediated by three hinges. Science.

[49] Hutchings J., Stancheva V., Miller E.A., Zanetti G. (2018). Subtomogram averaging of COPII assemblies reveals how coat organization dictates membrane shape. Nat Commun.

[bib50] Maeots M.E., Lee B., Nans A., Jeong S.G., Esfahani M.M.N., Ding S., Smith D.J., Lee C.S., Lee S.S., Peter M. (2020). Modular microfluidics enables kinetic insight from time-resolved cryo-EM. Nat Commun.

[bib51] Dandey V.P., Budell W.C., Wei H., Bobe D., Maruthi K., Kopylov M., Eng E.T., Kahn P.A., Hinshaw J.E., Kundu N. (2020). Time-resolved cryo-EM using Spotiton. Nat Methods.

[52] Frank J. (2017). Time-resolved cryo-electron microscopy: recent progress. J Struct Biol.

[53] Rosenthal P.B., Henderson R. (2003). Optimal determination of particle orientation, absolute hand, and contrast loss in single-particle electron cryomicroscopy. J Mol Biol.

[54] Himes B.A., Zhang P. (2018). emClarity: software for high-resolution cryo-electron tomography and subtomogram averaging. Nat Methods.

[55] Bartesaghi A., Lecumberry F., Sapiro G., Subramaniam S. (2012). Protein secondary structure determination by constrained single-particle cryo-electron tomography. Structure.

[56] Song K., Shang Z., Fu X., Lou X., Grigorieff N., Nicastro D. (2020). In situ structure determination at nanometer resolution using TYGRESS. Nat Methods.

[57] Sanchez R.M., Zhang Y., Chen W., Dietrich L., Kudryashev M. (2020). Subnanometer-resolution structure determination in situ by hybrid subtomogram averaging - single particle cryo-EM. Nat Commun.

